# An Initiative for the Study and Use of Genetic Diversity of Domesticated Plants and Their Wild Relatives

**DOI:** 10.3389/fpls.2018.00209

**Published:** 2018-02-20

**Authors:** Alicia Mastretta-Yanes, Francisca Acevedo Gasman, Caroline Burgeff, Margarita Cano Ramírez, Daniel Piñero, José Sarukhán

**Affiliations:** ^1^CONACYT – Comisión Nacional para el Conocimiento y Uso de la Biodiversidad, Mexico City, Mexico; ^2^Comisión Nacional para el Conocimiento y Uso de la Biodiversidad, Mexico City, Mexico; ^3^Instituto de Ecología, Universidad Nacional Autónoma de México, Mexico City, Mexico; ^4^Instituto de Ecología, Comisión Nacional para el Conocimiento y Uso de la Biodiversidad, Universidad Nacional Autónoma de México, Mexico City, Mexico

**Keywords:** Mexico, food security, food sovereignty, milpa, forestry, agroecology, conservation genetics

## Abstract

Domestication has been influenced by formal plant breeding since the onset of intensive agriculture and the Green Revolution. Despite providing food security for some regions, intensive agriculture has had substantial detrimental consequences for the environment and does not fulfill smallholder’s needs under most developing countries conditions. Therefore, it is necessary to look for alternative plant production techniques, effective for each environmental, socio-cultural, and economic conditions. This is particularly relevant for countries that are megadiverse and major centers of plant domestication and diversification. In this white paper, a Mexico-centered initiative is proposed, with two main objectives: (1) to study, understand, conserve, and sustainably use the genetic diversity of domesticated plants and their wild relatives, as well as the ongoing evolutionary processes that generate and maintain it; and (2) to strengthen food and forestry production in a socially fair and environmentally friendly way. To fulfill these objectives, the initiative focuses on the source of variability available for domestication (*genetic diversity* and *functional genomics*), the context in which domestication acts (*breeding* and *production*) and one of its main challenges (*environmental change*). Research on these components can be framed to target and connect both the theoretical understanding of the evolutionary processes, the practical aspects of conservation, and food and forestry production. The target, main challenges, problems to be faced and key research questions are presented for each component, followed by a roadmap for the consolidation of this proposal as a national initiative.

## Evolution Under Domestication Faces Modern Challenges

Species’ domestication is an evolutionary process in which humans, by means of artificial selection, take advantage of the genetic diversity of a wild species and modify it to our needs (Darwin, 1859; Casas et al., 2016). The domestication of plants started around 10,000 years ago, first for food production and then for forestry. Domesticating plants led to the independent invention of agriculture by several cultures around the globe and the emergence of ‘agrobiodiversity’ (Diamond, 2012). The process of domestication is ongoing, and today occurs in a wide range of systems that span traditional farming to industrialized large-scale agriculture. These, and all forms of farming, have a common challenge: how to feed humankind in the future within a context of food sovereignty and climate change, while conserving people’s biocultural legacy and the remaining natural ecosystems of Earth.

Domestication was particularly modified by formal plant breeding after World War II, with the onset of intensive agriculture (Harlan, 1975). This type of agriculture was then introduced to developing countries during the ‘Green Revolution’ (1960–1990). In this period, food security was treated as an issue of increasing production through breeding elite cultivars under conditions of high inputs (e.g., fertilizers and pesticides), and selecting for higher yields, wide (instead of local) adaptation, and adaptability to mechanical harvest technologies (Sonnenfeld, 1992; Crow, 1998; Baranski, 2015). All these considerably increased total yields of a small number of grain species, which allowed for dramatically increases in food production and lower global food prices. However, high-input agriculture, promoted by the Green Revolution, also had important detrimental consequences and limitations (Tilman, 2001; Evenson and Gollin, 2003), among them: First, elite cultivars work well only in high quality soil, with high water availability and intensive use of inorganic fertilizers (Duvick, 2005; Ceccarelli, 2009). Second, it promotes species- and genetic-homogeneity, making cultivars vulnerable to pests and diseases (Ullstrup, 1972), thus making necessary the use of pesticides in an “arms race” (Després et al., 2007). The heavy use of these fertilizers and pesticides is also harmful to the wider environment. Examples of this damage are the vast marine “dead zones” that exist at every coastline where rivers coming from intensive agriculture areas meet the ocean (Rabalais et al., 2010). Third, breeding for intensive agriculture switched the domestication process from the farmers to researchers and commercial seed companies (Troyer, 2009; Ceccarelli, 2015). As a result, many local species and varieties were abandoned (Ceccarelli, 2009); the capacity to keep, generate, and apply traditional agro-knowledge started to disappear (Gómez-Baggethun and Reyes-García, 2013); and the food security of several areas became dependent on a decreasing number of crops (Khoury et al., 2014), that are increasingly controlled by a few agro-industrial companies (Howard, 2009). Lastly, focusing artificial selection only on yield led to deficiencies in micronutrients (FAO, 2010).

These unintended consequences cannot be ignored, especially in developing countries that still hold important remnants of natural ecosystems and native agrobiodiversity. Also, given the diversity of environments and social conditions where agriculture occurs in such ecologically diverse countries, it is highly unlikely that any single agricultural system will solve their problems of food and fiber production (Kahane et al., 2013). Therefore, we should re-think the path we have been taking in relation to modern-day domestication, and look for alternatives that are effective for each environmental, socio-cultural and economic context.

## The Initiative

In this white paper we describe a Mexico-centered initiative with two objectives: (1) to study, understand and conserve the genetic diversity of native crops and their wild relatives, and preserve the ongoing domestication processes that generate and maintain this diversity; and (2) to use this diversity to strengthen food and forestry production in a socially fair and environmentally friendly way. These objectives relate to biodiversity conservation because the fate of the remaining ecosystems of Earth depends on how we undertake agricultural and forestry production over the following decades (Tscharntke et al., 2012); moreover, they are relevant to food sovereignty because the rights of peoples to healthy and culturally appropriate food depends on our ability to conserve and effectively use domesticated species. These objectives are relevant to Mexico, one of the Vavilov Centers for plant domestication (Vavilov, 1951), because it is a megadiverse country where smallholders of a variety of cultural groups practice agriculture and forestry in a diversity of agricultural systems and environments. However, the core of this initiative should be useful for similar countries.

This initiative proposes going back to two core elements of domestication: (1) the genetic diversity of domesticated species, their wild relatives and associated microbiome, and (2) the evolutionary potential that implies having millions of smallholders cultivating extensive areas of diverse crops in different environments. These are core elements for the following reasons.

First, genetic diversity provides options to grow diverse and nutritious food with fewer resources, adapted to harsher environments, and making cultivars less susceptible to pests and diseases. Proof of this is that cultivars can already be grown in a wide variety of environments, including marginal conditions where commercial lines do not perform well (Ceccarelli, 2009; Dwivedi et al., 2016). Similarly, crops’ wild relatives tend to have higher genetic diversity in terms of drought, pest, and disease resistance than their cultivated counterparts (Maxted et al., 2013). To that diversity, we can add an even larger set of microorganisms that have co-evolved with these species and their environment. This microbiome can greatly influence plant performance, but further applied research is needed (Sessitsch and Mitter, 2015; Gopal and Gupta, 2016; Qin et al., 2016; Benitez et al., 2017).

Second, to take full advantage of and maintain the evolutionary processes that generated this diversity, we should change our vision to vindicate the role of smallholder farmers (<5 ha) not only as a productive force, but also as an un-substitutable engine for crop evolution under diverse and challenging environments. Crop genetic diversity is not useful by itself: it needs to be related to production practices that can make the most out of the traits given by genetic diversity. The people who use this genetic diversity and possess its associated traditional knowledge are the heirs of the domestication processes that indigenous groups started 1000s of years ago (Boege, 2008). They tend to be smallholder *campesinos* that keep their own seed and devote part of their production for self-sufficiency. Although they are commonly seen as ‘unproductive,’ they are the backbone of food security (FAO, 2014). Similarly, the generation and maintenance of crop genetic diversity depends on millions of smallholders cultivating under different environmental conditions and cultural preferences which, from an evolutionary perspective, represent the best way to maintain and generate genetic diversity (Enjalbert et al., 2011; Perales, 2016; Comisión Nacional para el Conocimiento y Uso de la Biodiversidad [CONABIO], 2017).

## Components

The initiative focuses on five components that span the source of variability available for domestication (*genetic diversity* and f*unctional genomics*), the context in which domestication acts (*breeding* and *production*) and one of the main challenges it faces (*environmental change*). The genetic diversity within crops’ wild relatives, cultivated species, and their associated microbiome is the basis of domestication. Functional genomics represents a second layer of information that allows mapping genetic diversity to useful traits, both in human and environmental terms. Genetic drift, linkage disequilibrium, and epigenetics also play an important role in shaping diversity among populations and within genomes. These types of data can help to understand and monitor domestication and environmental adaptation (Gepts, 2014; Lasky et al., 2015). However, to conserve and use this diversity to adapt crops to environmental change, it is also necessary to consider the context in which the evolutionary forces of domestication act (Enjalbert et al., 2011). This context includes the biocultural and environmental factors that are given by breeding and production.

The Mexican context and the initiative’s aim by component are summarized below, and Supplementary Table S1 shows challenges and key research questions.

### Genetic Diversity

Mexico has ∼280 native plant species with forestry potential (FAO, 2011) and more than 130 that are used as food sources. Among the latter are maize, beans, pumpkins, chili, amaranths, vanilla, and 20 more that are of high economic importance worldwide (Acevedo et al., 2009). Mexico is the center of domestication or diversification of these and several more crop species (Acevedo et al., 2009). There are also potentially 1000s of wild species related to them, as shown by the existence of ∼270 wild relatives belonging to the gene pool of the 12 main Mexican crops (CONABIO and UICN, 2016). Within each domesticated species there are dozens of varieties (e.g., 59 maize landraces, and 60 chili types; Aguilar-Rincón et al., 2010; Comisión Nacional para el Conocimiento y Uso de la Biodiversidad [CONABIO], 2011). The microbiome of these species has only just started to be explored, but it is likely very large. This large diversity of species, cultivars, microbes, and genes is not static: it is still evolving in a complex context of environmental conditions that range from sea level to cold highlands, and under the continuous domestication of 68 indigenous groups (Comisión Nacional para el Desarrollo de los Pueblos Indígenas [CDI], 2014) and *campesino* farmers.

**FIGURE 1:**
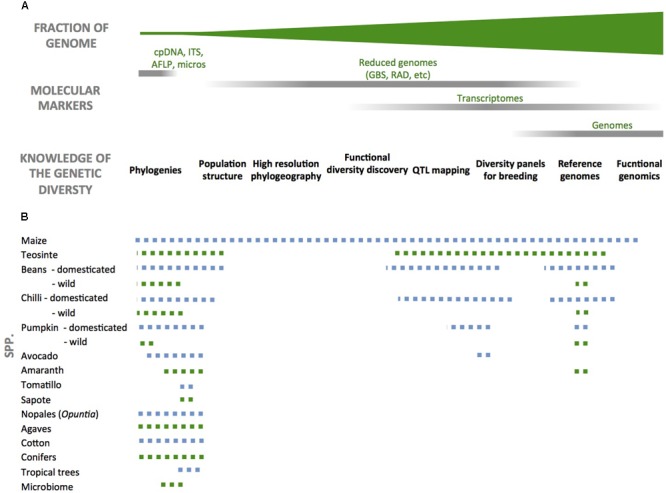
Current state of knowledge on the genetic diversity of Mexican crop and forestry species. **(A)** Top to bottom: fraction of the genome (green triangle) that is represented by different molecular markers (gray bars) and below the kind of knowledge these are used for. **(B)** Dashed lines represent the types of studies that have been developed for each species according to the markers and topics from the top. The size of the line is an approximate representation for the level of current knowledge for each species. Differences in line color are only for visual purposes.

There is a considerable body of research on key Mexican domesticated species like maize (e.g., Arteaga et al., 2016; Romero Navarro et al., 2017), but limited work for most of the rest (Bellon et al., 2009; Piñero et al., 2009; **Figure 1**). Similarly, maize landraces’ distribution and domestication history has been widely analyzed (Kato et al., 2009; Comisión Nacional para el Conocimiento y Uso de la Biodiversidad [CONABIO], 2011), but this information remains unknown or has not been systematized for the rest of the species. This initiative aims to study, conserve, evaluate, safe keep, analyze, and sustainably use this genetic diversity.

### Functional Genomics

From studies in functional genomics we know that the phenotypes obtained during the early stages of domestication are governed by relatively few genomic loci, which, in general, are different from the loci involved in later phenotypic diversification, and from the loci subjected to natural selection (Meyer and Purugganan, 2013). Research on these topics has mostly been conducted abroad, but Mexican research institutions devoted to these areas were recently created. In developing this research, it is important to consider native varieties and molecular markers representative of this variation (Ganal et al., 2011; Caldu-Primo et al., 2017).

This component aims to have well-annotated genomes and diversity panels for the main agriculture and forestry species of importance to Mexico, along with their wild relatives. This should allow for a better understanding of the molecular basis of domestication, but also to apply this information to breeding, conserving, and monitoring.

### Production

In Mexico, most agricultural and forestry land belongs to campesino or indigenous communities. Their agriculture tends to be performed in blocks <5 ha, which characterizes them as smallholders. Although they are often perceived as ‘unproductive,’ the campesinos’ aggregated production is the backbone of Mexico’s food security (Bellon et al., under review). However, since the 1980s, the programs directed at smallholder agriculture were drastically reduced, or redirected to provide farmers with Green Revolution packages (Turrent-Fernández and Cortés-Flores, 2005b). Remarkably, many smallholders continue to use traditional varieties (Eakin et al., 2014), because these are adequate and competitive when grown under their local conditions (Muñoz et al., 1976), and because smallholders not only focus on yield, but also look to fulfill cultural preferences (Brush and Perales, 2007; Bellon and Hellin, 2011).

Importantly, smallholder farmers tend to be able to obtain some degree of usable yield in underperforming environments without aid (Bellon et al., under review). This has two main implications. First, the productivity gap of many of these farmers could be closed with minor agronomic improvements and breeding, with important consequences. Second, millions of farmers, spread across a wide variety of environments and cultural preferences, represent the ideal scenario for evolution under domestication to continue to occur at the scale and range needed to effectively maintain and generate new genetic diversity.

This component aims attending agricultural and forestry production so that they can be increased in a sustainable and sovereign way, while also fulfilling the Mexican needs regarding desired cultivars, quantity, quality, and local cultural preferences. For this, it is crucial to protect smallholders’ production and target it with adequate programs that consider management, markets, and education.

### Plant Breeding

Formal plant breeding was introduced into Mexico in replication of the model of the United States. However, in Mexico most production does not use seeds generated by ‘formal’ breeding (Turrent-Fernández and Cortés-Flores, 2005a,b; Donnet et al., 2012). This could be interpreted as a failure for formal breeding, but also as the success of smallholders’ practices of domestication.

Commercial lines have not been widely adopted for several motives, one is that the formal breeding lines tend not to be useful to the smallholders’ production systems and environments. This is a consequence of the way breeding is performed and targeted. The environmental conditions characteristic of the areas where the vast majority of smallholders are located are poorly represented by the Mexican public research stations where improved lines have been developed (Bellon et al., 2005). Also, the selection goals of the smallholders can vary depending on the particular characteristics of the local environment and the producer’s interests, which do not necessarily focus on maximizing yield. For these reasons, in the areas and conditions where local varieties are traditionally grown, they tend to have a better performance than the improved lines in terms of yield, nutritional value, forage quality, local appreciated taste, or precocity (Muñoz et al., 1976; Perales et al., 1998; Sociedad Mexicana de Fitogenética [SOMEFI], 2007, 2009).

Given the range of conditions under which smallholders conduct agriculture and the limited success of formal breeding over the last 70 years in Mexico, it is unlikely that commercial-type breeding would become a successful strategy. Instead, Mexico should recognize, and incorporate into breeding, the diversity of traditional and local knowledge on shaping and adapting cultivars. For this, public policies should be shifted to better support informal breeding, and to focus formal breeding on the smallholders’ needs and adaptation to local conditions. For this, landraces should be incorporated as the base material of breeding programs, instead of only as donors for elite materials (Comisión Nacional para el Conocimiento y Uso de la Biodiversidad [CONABIO], 2017).

The aim for this component is to have breeding programs for a range of native species, using alternative tools to accelerate and improve the breeding process, where the objective would be breeding for the smallholders’ needs under present and future, social and environmental conditions. To accomplish this, alternative tools need to be explored, including documenting and sharing campesino-to-campesino experiences, participatory breeding, genomic selection and evolutionary breeding.

### Environmental Change

Mexico’s agriculture and forestry production are facing environmental change in the form of soil degradation, pollution, invasive species and climate change. It is difficult to generalize how environmental change would affect a particular crop or wild species; for instance, the effect of climate change on maize depends on the plant’s genotype, local environment, and management (Mercer and Perales, 2010). Nonetheless, we know that environmental change has important economic impacts. For example, ∼10% of Mexican agriculture land is eroded, which translates into ∼50% of PROCAMPO aid costs (Sánchez-Colón et al., 2009; Ávalos et al., 2011). Given the large diversity of environments where Mexican crop and wild species occur, it is likely that useful variation to cope with new sources of stress already exists. What is needed is to make that diversity available to producers by enhancing seed-exchange networks, breeding and access to seeds, and environmental information at local and national scales. Therefore, this component aims to accelerate the adaptation of cultivated plants to environmental change and look for effective mechanisms to conserve the capacity of wild populations to adapt.

## Roadmap

Research on the previous components could target and connect both the theoretical understanding of the evolutionary processes, and the practical aspects of applying this knowledge to conservation and production. However, for this to happen in a National scale, it is necessary to systematize and make data available to both the academy and wider public, and to influence public policy. CONABIO is a Mexican inter-ministerial commission in charge of that type of activities regarding biodiversity. We therefore envision that CONABIO would help to develop the initiative’s in the following early stages:

(1) Pilot research projects on genetic diversity of Mexican cultivars and wild relatives, developed by CONABIO and external research groups.
(2) An information system developed by CONABIO that allows data on agrobiodiversity and genetic diversity to be analyzed, archived, and made public.
(3) Better and more collaboration between research groups, civil organizations and education institutions to accelerate participatory research and the formation of human resources.
(4) Implementation of public policy recommendations and participatory research congruent with the Mexican reality and smallholders needs.

Examples of the first steps are already ongoing. For instance, in the case of wild relatives, a systematic conservation planning analysis is being performed incorporating both the distribution of genetic diversity and social variables^1^. For the cultivated forms, maize is the species with more data available on the distribution of native races and their genetic characterization. What is next needed is to integrate its management practices, uses and environmental and microbiome data, to then make this information available and accessible to farmers, breeders and wider audience. The target of this should be to strengthen seed exchange networks, resources for participatory breeding and campesino-to-campesino experience sharing. Molecular tools would help to accelerate breeding, especially at the stage of crosses design and genetic diversity monitoring. We estimate that with minimal breeding support and agronomic improvements, it would be possible to increase the average yield of 4 million ha from 1.3 to 2.3 ton/ha. This would be enough to cover the maize needs of c.a. 88.5 million people (Comisión Nacional para el Conocimiento y Uso de la Biodiversidad [CONABIO], 2017) without implementing intensive agriculture systems.

## Author Contributions

AM-Y, MCR, and CB performed literature reviews and contributed to the discussion. AM-Y, DP, FAG, and JS wrote the manuscript. All authors conceived and designed the manuscript.

## Conflict of Interest Statement

The authors declare that the research was conducted in the absence of any commercial or financial relationships that could be construed as a potential conflict of interest. The handling Editor declared a shared affiliation, though no other collaboration, with the authors.

## Footnotes

1 http://www.psmesoamerica.org/en/
