# Supplementary material for: An Initiative for the Study and Use of Genetic Diversity of Domesticated Plants and Their Wild Relatives
Source: Front Plant Sci. 2018 Feb 20;9:209. doi: 10.3389/fpls.2018.00209 (PMC5826304; doi:10.3389/fpls.2018.00209)
Supplement: Supplementary file 1 [file Table_1.PDF]

Table S1. Identified challenges and key research questions for each component of the initiative

Component	Problems and challenges	Research questions
Genetic Diversity	● Genetic diversity is being lost due to habitat destruction, inadequate farm management, reduction the farmer populations, and increased use of genetically homogenous cultivars<br>● Genetic diversity conservation programs (in situ and ex situ) are neither well defined nor follow a concrete strategy<br>● The impact and success of current genetic conservation programs (ex situ and in situ) have not been evaluated<br>● The potential of genetic diversity is underused<br>● There is not an integrative information system to systematically collect and access data and knowledge on the genetic diversity of Mexican crops, forestry species and their microbiome.	1. What are the actual distributions of domesticated plants, their variants and wild relatives and which areas have not yet been sampled?<br>2. What are the temporal and spatial distributions of genetic diversity within the domesticated plants and their wild relatives?<br>3. How much genetic diversity is there at the population level?<br>4. Which geographic areas can be identified as hotspots of species and genetic diversity for each of the domesticated taxa and their wild relatives?<br>5. What is the taxonomic and metabolic diversity of the microbiome associated to Mexican cultivars?<br>6. What are the effects of management, production and seed exchange practices on the genetic structure, amount of variation and the spatial distribution of domesticated species?<br>7. Which conservation programs (*ex situ* and *in situ*) successfully conserve genetic diversity?
Functional Genomics	● There are genomic resources (e.g. reference genomes, diversity panels) for only a few cultivars of international importance (Fig. 2), but not for the rest of the species and varieties of importance for Mexico	1. How much functional variation is there in the genetic diversity of populations/accessions of Mexican domesticated species and their wild relatives?<br>2. What are the genetic bases of the adaptation

	● There are few people professionally trained in functional genomics<br>● There is little or no connection between basic research and its application to breeding, production and small scale farming.	to different environmental conditions, pests and diseases within domesticated plants and their wild relatives?<br>3. How does domestication and artificial selection shape crop genomes?<br>4. Which regions within genomes are useful candidates to face changes in environmental conditions?<br>5. How do management and production practices (including interactions with wild-relatives) affect the genomic composition of domesticated species?
Production	● Public investment in agriculture has been directed to large producers with an agro-industrial focus, and not to the smallholders that represent the majority of Mexican producers<br>● Aid programs are homogeneous whilst the needs and types of producers are heterogeneous<br>● Aid programs for smallholders are focused on social subsides and not production<br>● Aid programs for production do not promote conservation of genetic resources<br>● The smallholder farmer population is ageing or migrating<br>● Mexican forests diversity is not used to its full potential by forestry<br>● The production potential of some areas could be improved with small management interventions, but these need to be determined case by case	1. What are the current and potential uses (traditional, gastronomic, industrial, among others) of agrobiodiversity and the products derived from it?<br>2. How does the microbiome affect production in different environments and management systems?<br>3. How does socioeconomic conditions, public policy and management favor or limit sustainable production at the local, regional and national levels?<br>4. Which local management conditions are socially adequate and contribute to improving producers' life conditions?<br>5. Is agrobiodiversity positively associated with the food security of the people safeguarding it?<br>6. What is the role of agrobiodiversity for the culture and subsistence of rural areas?<br>7. How to effectively make case-by-case management recommendations to smallholders considering their wide national distribution?

Breeding	● There is a lack of breeding efforts for many cultivars and traits that are important for Mexico from a cultural or gastronomic perspective<br>● Most breeding efforts focus on wide adaptation and high-inputs and few promote local adaptation to marginal environments<br>● Few breeding efforts focus on improving native materials for local environmental and social conditions.<br>● New tools (evolutionary, genomic, participative) are not being incorporated to accelerate and improve breeding<br>● There are few people professionally formed in plant breeding<br>● Campesino-to-campesino and informal breeding experiences are not supported.	1. What are the management and breeding needs of the smallholders considering both present and future conditions under a scenario of climate change?<br>2. In which traits useful for adverse environmental conditions should breeding focus on at local and regional levels?<br>3. What are the opportunities and limitations of using new tools (genomic, participative, evolutionary) for plant breeding in Mexico?<br>4. Under what conditions can participatory breeding contribute to the conservation of genetic resources and empowerment of rural communities?<br>5. Which strategies should be followed to resolve the agronomic needs of smallholders?<br>6. How can informal breeding (farmer selection, seed exchange) be supported effectively?
Environmental change	● There are no high resolution maps for soil erosion and pollution, and global climate change models cannot predict accurately and at high spatial resolution what the future climate conditions might be like<br>● Climate change is a threat to all cultivated plants and their wild relatives, independently of type of management and production<br>● Current practices may not be enough to provide farmers with the variation needed to cope with environmental change<br>● Environmental change is occurring faster	1. What are the current environmental conditions where the species of interest are cultivated and where do their wild relatives exist?<br>2. What would be their potential distribution under climate change conditions?<br>3. Which genotypes within every domesticated and wild relative species are adapted to which of the diverse environmental conditions of Mexico?<br>4. Are there traditional practices associated to the adaptation of cultivars to harsh environmental conditions?<br>5. Are there in Mexico cultivated plants under

	than the natural capacity of species to adapt	environmental conditions that would be analog to the predicted conditions of environmental change for other parts of the country?<br>6. Can seed exchange and informal breeding be assisted to provide smallholder farmers with the variation needed to cope with environmental change?
